# S⋯N Conformational Lock Acceptor Based on Indacenodithiophene (IDT) Structure and High Electronegative Terminal End Group

**DOI:** 10.3390/ma15124238

**Published:** 2022-06-15

**Authors:** Jiejun Zhu, Zhangxu Wang, Yuanhao Li, Xuan Liu, Chunyang Miao, Bo Wu, Shiming Zhang

**Affiliations:** 1 Department of Physics, Nanjing Tech University, 30 South Puzhu Road, Nanjing 211816, China; zhujj@njtech.edu.cn; 2 Key Laboratory of Flexible Electronics (KLOFE) & Institute of Advanced Materials (IAM), Jiangsu National Synergetic Innovation Center for Advanced Materials (SICAM), Nanjing Tech University (NanjingTech), 30 South Puzhu Road, Nanjing 211816, China; 201961122074@njtech.edu.cn (Z.W.); 202061222186@njtech.edu.cn (Y.L.); 202061122027@njtech.edu.cn (X.L.); 3 Jiangsu Seenbom Flexible Electronics Institute Co., Ltd., Building 5, Room 201, 6 Zhida Road, Nanjing 210043, China

**Keywords:** organic photovoltaics, end group, electronegativity, energy gap, electron-rich

## Abstract

High-performance organic semiconductors should have good spectral absorption, a narrow energy gap, excellent thermal stability and good blend film morphology to obtain high-performance organic photovoltaics (OPVs). Therefore, we synthesized two IDTz-based electron acceptors in this research. When they were blended with donor PTB7-Th to prepare OPV devices, the PTB7-Th:IDTz-BARO-based binary OPVs exhibited a power conversion efficiency (PCE) of 0.37%, with a short-circuit current density (*J*_sc_) of 1.24 mA cm^−2^, a fill factor (FF) of 33.99% and an open-circuit voltage (*V*_oc_) of 0.87 V. The PTB7-Th:IDTz-BARS-based binary OPVs exhibited PCE of 4.39%, with *J*_sc_ of 8.09 mA cm^−2^, FF of 54.13% and *V*_oc_ of 1.00 V. The results show the strong electronegativity terminal group to be beneficial to the construction of high-performance OPV devices. Highlights: (1) Two new acceptors based on 5,5′-(4,4,9,9-tetrakis (4-hexylphenyl)-4,9-dihydro-s-indaceno [1,2-b:5,6-b′] dithiophene-2,7-diyl) dithiazole (IDTz) and different end groups (BARS, BARO) were synthesized; (2) BARS and BARO are electron-rich end groups, and the electron acceptors involved in the construction show excellent photoelectric properties. They can properly match the donor PTB7-Th, and show the appropriate surface morphology of the active layer in this work; (3) Compared with IDTz-BARO, IDTz-BARS has deeper LUMO and HOMO energy levels. In combination with PTB7-Th, it shows 4.39% device efficiency, 8.09 mA cm^−2^ short-circuit current density and 1.00 V open circuit voltage.

## 1. Introduction

In recent years, bulk heterojunction structure organic photovoltaics (OPVs) have become a research hotspot in new energy studies because of their low cost [1], solution processability [2] and device flexibility [3,4] for large-area and high-volume production [5]. Over the years, from the ITIC structure [6] and their derivatives [7,8,9] to Y6 [10] and Y6 derivatives [11,12,13], the narrow band gap non-fullerene acceptors (NFAs) were found to have excellent OPV device performance [14,15,16], with the result that the maximum efficiency of organic solar cells constantly updated. At present, the power conversion efficiency (PCE) of OPV devices based on Y6 derivatives as the acceptor has exceeded 17% [17,18], which exhibits promising prospects for industrialization.

High-performance OPV devices mainly come from molecular acceptors with high charge transport mobility, which are usually small molecular acceptors with high conjugation planes [19,20]. In recent years, non-fullerene electron acceptors have been continuously developed to improve the device efficiency of OPVs to satisfactory performance [14]. However, high-planar-conjugated aromatic structures are complex in organic synthesis, and a large number of synthesis steps may reduce cost-effectiveness, affecting the further industrialization of OPVs [21]. In 2012, Huang et al. [22] proposed the concept of conformational lock, in which a non-covalent bond was introduced into the acceptors of organic solar cells for the first time. This method promoted the synthesis of high-performance planar-conjugated electron acceptors [23,24]. Subsequently, Cao et al. [25] reported that 4,9-dihydro-sindaceno [1,2-b:5,6-b′]-dithiophene (IDT) was the core structural unit, and F-substituted benzothiadiazole was the electron acceptor of the bridging unit. At the same time, some reports had used non-covalent conformational locks (NoCLs), such as S⋯O [26,27,28], S⋯N [29,30] and Se⋯O [31], to construct molecular structures. Since then, NoCLs have played an important role in the design and synthesis of high-performance organic semiconductors [32]. In addition, Guo et al. reported a high-performance n-type semiconductor using F⋯H and F⋯S in their work on organic thin-film transistors (OTFTs) [33]. A variety of NoCLs have gradually become some of the principal ways to construct electron acceptors [34]. Specifically, in the work of Huang et al., the carbon atom in the original molecule was replaced with a nitrogen atom, resulting in a thiazole unit that induces N-S non-covalent conformation lock, with the goal of obtaining high-performance electron acceptors. Compared with Zhan et al.’s work in 2015 [6], Huang et al.’s work in 2017 [24] replaced the thiophene unit in IDT-T with thiazole, designed and synthesized the non-fullerene small-molecule acceptor IDT-Tz, and improved the device performance from 4.1% to 8.4% after mixing with donor PTB7-Th. Therefore, in this research, we employed this non-covalent construction method to develop new narrow-band-gap, non-fullerene electron acceptors to further expand the device performance of OPVs.

**Scheme 1:**
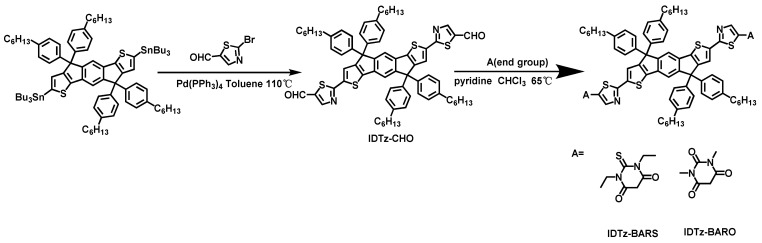
Synthetic route to IDTz-BARS and IDTz-BARO in this work.

The electron acceptor IDTz, based on IDT core and thiazole, achieved 8.4% PCE when PTB7-Th was used as the electron donor, which is higher than the device performance of an electron acceptor based on IDT core and thiophene. However, the ultraviolet–visible spectroscopy (UV-Vis) absorption peaks of IDTz-based electron acceptors 2,2′-((2Z,2′Z)-(((4,4,9,9-tetrakis (4-hexylphenyl)-4,9-dihydro-s-indaceno [1,2-b:5,6-b′] dithiophene-2,7-diyl) bis (thiazole-2,5-diyl)) bis (methaneylylidene)) bis (3-oxo-2,3-dihydro-1H-indene-2,1-diylidene)) dimalononitrile (IDT-Tz) and 2-((E)-5-((2-(7-(5-((E)-((Z)-2-(cyano (isocyano) methylene)-3-ethyl-4-oxothiazolidin-5-ylidene) methyl) thiazol-2-yl)-4,4,9,9-tetrakis (4-hexylphenyl)-4,9-dihydro-s-indaceno [1,2-b:5,6-b′]dithiophen-2-yl) thiazol-5-yl) methylene)-3-ethyl-4-oxothiazolidin-2-ylidene) malononitrile (IDTzCR) in solution were 649 and 603 nm, respectively, lower than that of ITIC (664 nm). In this work, we synthesized two A-π-D-π-A non-fullerene small-molecule acceptors, in which IDT was used as a D unit and thiazole as a π bridge unit. Known building blocks 1,3-diethyl-2-thiodihydropyrimidine-4,6 (1H, 5H)-dione (BARS) [35] and 1,3-dimethylpyrimidine-2,4,6 (1H, 3h, 5H)-trione (BARO) [36] monomers participated in the molecules as A units. We synthesized a series of OPV electron acceptors IDTz-BARS and IDTz-BARO with IDTz and different end groups. The molecular structure is shown in Scheme 1. The acceptors were mixed with donor PTB7-Th to prepare OPVs. The related properties and device results show that BARS exhibited excellent performance as the end group unit of IDTz structure in this work.

Compared with the ITIC, the UV-Vis absorption spectrum of the IDTz structure was blue-shifted. We anticipate that a satisfactory electron acceptor structure can be constructed through appropriate end group building blocks to obtain wider spectral absorption and a narrower optical bandgap (*E*_g_^opt^).

## 2. Materials Synthesis

The two chemical structures and synthetic routes of the new acceptors IDTz-BARS and IDTz-BARO are shown in Scheme 1. In recent years, the Knoevenagel reaction, which combines aldehyde group and end group units, has been widely used in the synthesis of organic semiconductors, and will likely continue to be used in organic synthesis in the future. In this work, the 2,2′-(4,4,9,9-tetrakis(4-hexylphenyl)-4,9-dihydro-s-indaceno [1,2-b:5,6-b′] dithiophene-2,7-diyl) bis (thiazole-5-carbaldehyde) (IDTz-CHO) and end group (BARS or BARO) were added to the reaction system, and pyridine was added to the mixture as a catalyst. Meanwhile, the synthetic route of IDTz-CHO in this research refers to the report of Huang et al. [25]. The intermediate IDT-SnBu_3_ was purchased from a commercial organization. These compounds were characterized by ^1^H-NMR and elemental analysis (Test data was shown in Figures S1–S3).

## 3. Results and Discussion

### 3.1. Optical and Thermal Property of Acceptors

**Figure 1:**
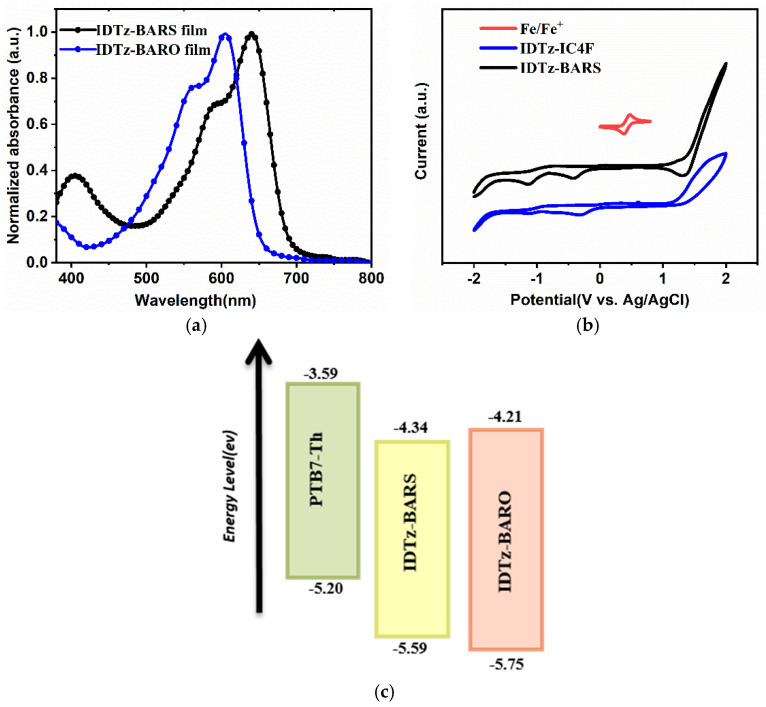
(**a**) Optical absorption spectra of IDTz-BARS and IDTz-BARO films spin-coated from chloroform solutions (10^−3^ M). (**b**) CV curves of acceptors IDTz-BARS and IDTz-BARO. (**c**) Energy levels of IDTz-BARS and IDTz-BARO.

The UV-Vis absorption spectra of IDTz-BARS and IDTz-BARO as thin films (spin-coated from 1 × 10^−3^ M CF solutions) are shown in Figure 1a. The relevant data, including absorption maximum (λ_max_), absorption edge (λ_onset_) and optical bandgap (*E*_g_^opt^), are shown in Table 1. In the film, the maximum absorption peaks of the UV-Vis spectra of IDTz-BARO and IDTz-BARS were 598 nm and 624 nm, respectively.

Compared with IDTz-BARO, the spectral absorption in the film of IDTz-BARS was red-shifted by 26 nm, indicating a strong intermolecular-packing solid state [37]. In Figure 1a, the absorption boundary of IDTz-BARS is 691 nm and the optical bandgap is 1.79 eV, which is 0.07 eV lower than that of IDTz-BARO (1.86 eV). The HOMO levels of IDTz-BARS and IDTz-BARO are −5.59 eV and −5.75 eV, respectively. The more electron-rich end group unit of BARS brings a higher HOMO energy level and narrower *E*_g_^opt^. This narrower band gap led to better exciton dissociation, with IDTz-BARS showing the highest *J*_sc_ [38]. The following formula was used to calculate the data in this work: *E*_g_^opt^ = 1240/*λ*_onset_ eV, *E*_LUMO_ = *E*_HOMO_ + *E*_g_^opt^ and *E*_HOMO_ = −*e* (*E*_ox_^onset^ + 4.80) eV, with the results shown in Table 1. In addition, the absorption red shift and wider absorption range of IDTz-BARS film beyond 600 nm, as well as the full-coverage absorption between 400 and 700 nm, show IDTz-BARS’s capture ability of photons. The absorption results show that BARS and BARO, as the end group unit of the molecule, can significantly reduce the band gap of A-D-A-type NFA molecules based on IDTz. Compared with IDTz-BARO, the energy level of IDTz-BARS indicates that it may be a high-performance electron acceptor.

**Figure 2:**
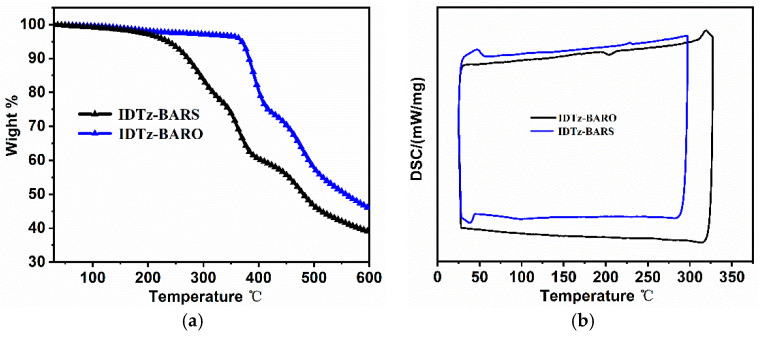
(**a**) Thermogravimetric analysis of acceptors IDTz-BARS and IDTz-BARO at a temperature ramp rate of 10 °C/min under N_2_. (**b**) DSC thermograms of IDTz-BARS and IDTz-BARO from the second heating and cooling scans at a temperature ramp rate of 10 °C/min under N_2_.

The stability of the acceptor was determined by thermogravimetric analysis (TGA) in N_2_, where the weight loss of the semiconductor was 5%. Under these conditions, IDTz-BARS and IDTz-BARO showed stability up to 238.0 °C and 378.0 °C, respectively. Data from thermogravimetric analysis (TGA) experiments are shown in Figure 2a. According to the TGA results, the higher decomposition temperatures of IDTz-BARO may offer a wider annealing operation window in the subsequent preparation of OPV devices, while there were some deficiencies in the thermal stability of IDTz-BARS. According to the report, the main reason for the poor thermal stability of IDTz-BARS may be the insufficient thermal stability of the end group [35]. Changing the annealing temperature of devices is one important method to improve the performance of electronic devices. High thermal stability can render IDTz-BARS and IDTz-BARO promising excellent semiconductor materials for OPVs. Differential scanning calorimetry (DSC) spectra showed that IDTz-BARS has a glass transition near 45 °C, while IDTz-BARO exhibited glass transitions near 190 °C, with the result that the devices annealed at 200 °C had a more stable film state, which improved their charge transfer efficiency.

### 3.2. Electrochemical Properties

The electrochemical properties of IDTz-BARS and IDTz-BARO were studied by cyclic voltammetry (CV). Specifically, a layer of IDTz-BARS and IDTz-BARO film was attached to the glass carbon working electrode, the electrolyte solution was 0.1 M [n-Bu_4_N]^+^[PF_6_]^−^CH_3_CN and the CV spectrum was tested at a potential scan rate of 100 mV·s^−1^. Ferrocene/ferrocium (Fc/Fc^+^) was used as a standard in the test. The CV curves of IDTz-based acceptor are exhibited in Figure 1b. After the oxidation peak was measured, the HOMO energy levels of IDTz-BARO and IDTz-BARS were calculated as −5.75 eV and −5.59 eV, respectively, using *E*_HOMO_ = −*e* (*E*_ox_^onset^ + 4.80) eV. The higher HOMO energy level of IDTz-BARS is provided by the strong electron-absorbing end group and high planarity backbone in the molecule. Meanwhile, combined with UV-Vis absorption data and *E*_LUMO_ = *E*_HOMO_ + *E*_g_^opt^, the LUMO levels of IDTz-BARO and IDTz-BARS were −4.22 eV and −4.34 eV, respectively. As previously reported, the HOMO and LUMO levels of IDTz-BARS were deeper than those of IDTz-BARO [39]. From the results, the A-D-A-type NFA molecules can achieve a suitable band gap and absorption spectrum through different end group building blocks. Meanwhile, the appropriate LUMO level of the acceptor can bring enough exciton dissociation to the device, when cooperating with the donor, to obtain high device performance. NoCLs involved in the construction of NFAs have been widely used in OPVs. Due to the interaction of S⋯N, the adjacent molecules present a more planar skeleton, which can effectively promote charge transfer. At the same time, BARS and BARO have also been shown to effectively improve the performance of OPV.

### 3.3. Theoretical Calculation

**Figure 3:**
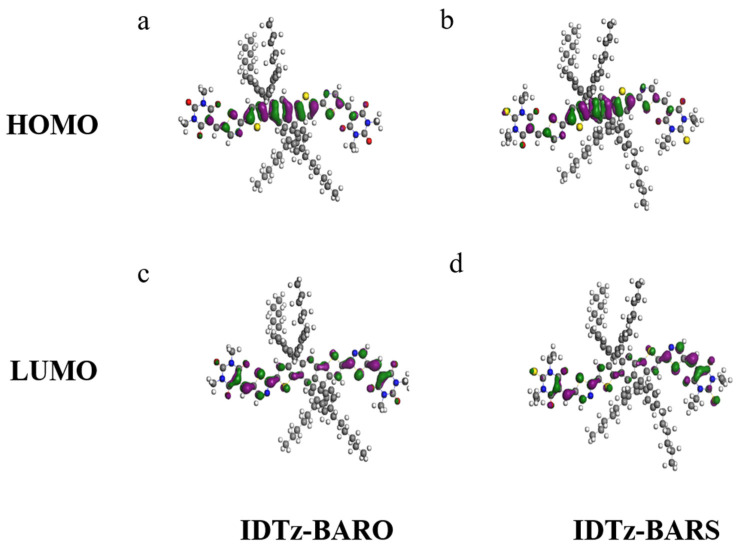
DFT calculation of HOMO energy levels of IDTz-BARO and IDTz-BARS (**a**,**b**). DFT calculation of LUMO energy levels of IDTz-BARO and IDTz-BARS (**c**,**d**).

To investigate the difference between the chemical geometry structure and the molecular frontier orbital of the small molecules, density functional theory (DFT) calculations were carried out with the G09 version D software package. Geometry optimizations were carried out with DFT methods B3LYP for geometry. Basis set 6-31G (d) was adopted for the C, H, O, N and S atoms, as shown in Figure 3. The calculation results show the energy level band gaps of IDTz-BARO (1.31 eV) and IDTz-BARS (1.24 eV). The HOMO levels of IDTz-BARO and IDTz-BARS were −4.90 eV and −4.86 eV, respectively. The LUMO levels of IDTz-BARO and IDTz-BARS were −3.59 eV and −3.62 eV, respectively. From the results, compared with IDTz-BARO, IDTz-BARS displays a smaller energy gap and higher HOMO and LUMO levels. As it is generally acknowledged that FMO energy levels of acceptor material are related to device performance, differences between the two acceptors were thus expected once embedded in active layers.

The DFT calculation results indicate that the HOMO energy level of IDTz-BARS was concentrated in the core of the molecular backbone, while the HOMO energy level of IDTz-BARO was dispersed within the whole molecular backbone. Compared with the near-identical LUMO levels of IDTz-BARO and IDTz-BARS, the LUMO level of IDTz-BARS was more concentrated in the end group units on both sides, and showed a lower LUMO level. From the results, IDTz-BARS shows the smallest energy level band gap in this work, in agreement with the UV-Vis absorption spectrum. Compared with IDTz-BARO, the narrower band gap is conducive to the excitation of electrons. In addition, the HOMO and LUMO levels of IDTz-BARS may be more easily combined with the donor material PTB7-Th (−4.86 eV and −3.62 eV) than that of IDTz-BARO and IDTz-BARS, ensuring effective exciton decomposition. In conclusion, the strong electron-withdrawing end group directed it towards lower HOMO and LUMO levels.

### 3.4. Film Morphologies and Organic Solar Cell Performance

**Figure 4:**
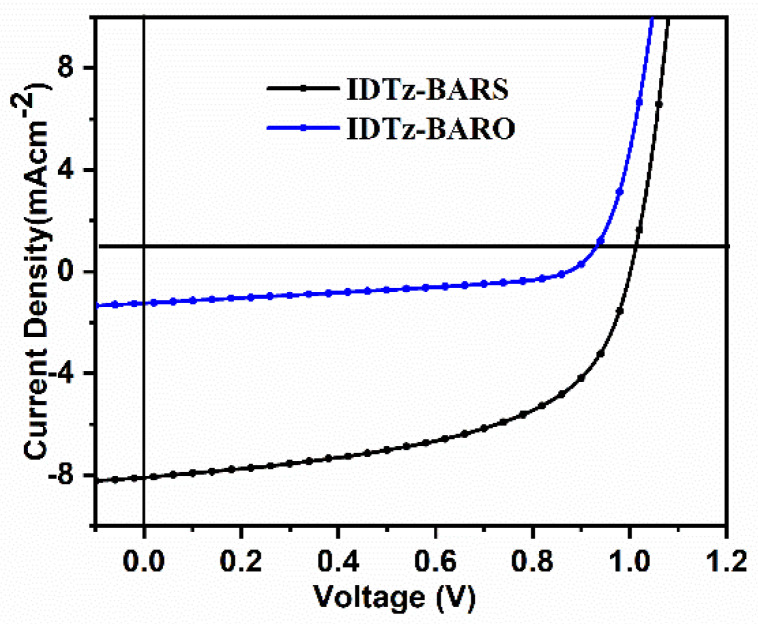
*J*–*V* curves of PTB7-Th:IDTz-BARO, PTB7-Th:IDTz-BARS and PTB7-Th.

In order to study the performance of materials in OPV devices, PTB7-Th was used as a donor, and the IDTz-BARS and IDTz-BARO were used as acceptors. The device structure was ITO/ZnO/donor (PTB7-Th):acceptor (IDTz-BARO or IDTz-BARS)/MoO_3_/Ag, and the inverted device structure was prepared with 1,2-dichlorobenzene (CB) as solvent. The current density–voltage (J–V) curves of PTB7-Th:IDTz-BARO and PTB7-Th:IDTz-BARS are shown in Figure 4, with corresponding data shown in Table 2. The binary OPVs based on PTB7-Th:IDTz-BARO had PCE of 0.37%, *J*_sc_ of 1.24 mA cm^−2^, FF of 33.99% and *V*_oc_ of 0.87 V. The PTB7-Th:IDTz-BARS-based binary OPVs exhibited PCE of 4.39%, with *J*_sc_ of 8.09 mA cm^−2^, FF of 54.13% and *V*_oc_ of 1.00 V.

The high performance of IDTz-BARS is mainly attributed to higher *J*_sc_ (8.09 mA cm^−2^ for IDTz-BARS and 1.24 mA cm^−2^ for IDTz-BARO). At the same time, the introduction of F atom strengthens the intermolecular force. Under the same device preparation conditions, the introduction of S atom is conducive to improving the FF of the active layer. In this work, when IDTz-BARO and IDTz-BARS were used in organic solar cells with PTB7-Th, the FF of the active layer was gradually increased (33.99% for IDTz-BARO, 54.13% for IDTz-BARS). The results reveal that the introduction of strong electron-absorbing end groups is an effective way to improve the *J*_sc_, FF and PCE of OPVs. It has been reported that the FMO energy level of organic semiconductors can affect the *V*_oc_ of materials in OPV [40]. Meanwhile, the *V*_oc_ values of IDTz-BARO and IDTz-BARS were 0.87 V and 1.00 V, respectively. The BARO end group reduces the LUMO level of the acceptor, so the electron acceptor with higher electronegativity shows the lowest *V*_oc_ in this work. Compared with IDTz-BARS, IDTz-BARO had more blue-shifted spectral absorption, so that the *J*_sc_ of OPVs with IDTz-BARO as the acceptor was smaller than that of IDTz-BARS as the acceptor.

**Figure 5:**
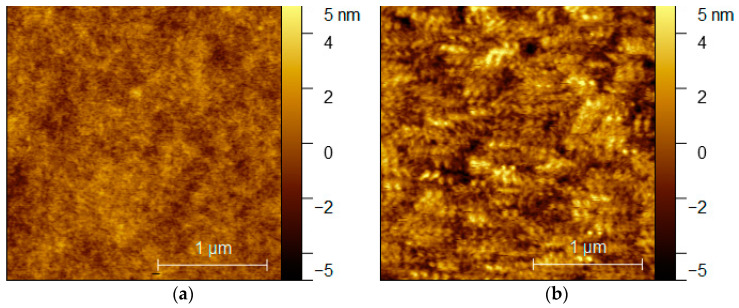
AFM characterization of (**a**) PTB7-Th:IDTz-BARO and (**b**) PTB7-Th:IDTz-BARS.

The AFM height images of PTB7-Th:acceptors are shown in Figure 5 (test area: 2.5 × 2.5 μm^2^), and the root mean square (RMS) roughness values of PTB7-Th:IDTz-BARO and PTB7-Th:IDTz-BARS were 0.79 nm and 1.50 nm, respectively. At the same time, the higher RMS surface roughness increases the contact area between the active layer and interfacial electrode, thus enhancing charge collection [41]. Among all the blends, PTB7-Th:IDTz-BARS showed the highest RMS roughness, which was likely attributed to IDTz-BARS having the strongest aggregation, as revealed by the UV-Vis absorption. Therefore, the AFM images indicate consistency in the absorption of IDTz-BARS and the performance of OPV devices.

## 4. Conclusions

Due to the introduction of S⋯N non-covalent conformation lock and thiazole as a π bridge, two IDTz-based non-fullerene acceptors were synthesized in this research. IDTz-BARS used BARS as the end group, while IDTz-BARO used BARO. Their solubility and thermal stability are sufficient for their application in OPV devices. IDTz-BARS had more red-shifted spectral absorption, a smaller energy gap and better film morphology, demonstrating a photoelectric conversion efficiency of 4.39% when cooperating with the donor PTB7-Th. Meanwhile, the two electron acceptors we synthesized have effective solubility and are easy to process in solution. The results show that the addition of two terminal groups without strong electron-withdrawing groups could also construct suitable electron acceptors. This work provides a promising strategy for the design of non-fullerene acceptors in non-covalent intramolecular conformational locks.

## Figures and Tables

**Table 1:** Optical absorption properties and electrochemical properties of IDTz-BARS and IDTz-BARO.

Molecule	λ_max_ (Sol) (nm)	λ_max_ (Film) (nm)	λ_onset_ (Film) (nm)	*E*_HOMO_ (eV)	*E*_LUMO_ (eV)	*E*_g_^opt^ (eV)
IDTz-BARO	598	605	665	−5.75	−4.22	1.86
IDTz-BARS	624	641	691	−5.59	−4.34	1.79

**Table 2:** Optimized photovoltaic performances of OPVs under illumination of AM 1.5 G irradiation (100 mW cm^−2^).

Molecule	Annealing ^a^ Temperature (°C)	*V_OC_* (V)	*J*_sc_ (mA cm^−2^)	*FF* (%)	PCE ^b,c^ (%)
IDTz-BARS	100	1.00	8.09	54.13	4.39 ± 0.33 (4.10)
IDTz-BARO	100	0.87	1.24	33.99	0.37 ± 0.05 (0.29)

^a^ Anneal at 100 ℃ for 5 min. ^b^ All weight ratios in this work are donor (PTB7-Th):acceptor (D/A) = 1:1.3. ^c^ The average PCE values were calculated using 10 separate devices and are shown in parentheses.

## Data Availability

Not applicable.

## Supplementary Materials

The following supporting information can be downloaded at: https://www.mdpi.com/article/10.3390/ma15124238/s1, Figure S1: ^1^H of compound 1 (r.t., in CDCl_3_); Figure S2: ^1^H of compound IDTz-BARO (r.t., in CDCl_3_); Figure S3: ^1^H of compound IDTz-BARS (r.t., in CDCl_3_).
