# Supplementary material for: S⋯N Conformational Lock Acceptor Based on Indacenodithiophene (IDT) Structure and High Electronegative Terminal End Group
Source: Materials (Basel). 2022 Jun 15;15(12):4238. doi: 10.3390/ma15124238 (PMC9227590; doi:10.3390/ma15124238)
Supplement: Supplementary file 1 [file materials-15-04238-s001.zip › materials-1731709-supplementary.pdf]

Supporting Information

S···N conformational lock acceptor based on indacenodithiophene (IDT) structure and high electronegative terminal end group

Jiejun Zhu,[a+] Zhangxu Wang,[b+] Yuanhao Li,[b] Xuan Liu,[b] Chunyang Miao,[b*] Bo Wu[b*] and Shiming Zhang[b,c*]

[a] *Department of Physics, Nanjing Tech University, 30 South Puzhu Road, Nanjing 211816, China*

[b] *Key Laboratory of Flexible Electronics (KLOFE) & Institute of Advanced Materials (IAM), Jiangsu National Synergetic Innovation Center for Advanced Materials (SICAM), Nanjing Tech University (NanjingTech), 30 South Puzhu Road, Nanjing 211816, China*

[c] *Jiangsu Seenbom Flexible Electronics Institute Co., Ltd. Building 5, Room 201, 6 Zhida road, Nanjing 210043, China*

+ These two authors contributed equally to this work

***Corresponding Author**

E-mail: iamsmzhang@njtech.edu.cn; iamcymiao@njtech.edu.cn; iambwu@njtech.edu.cn

Experimental Section

S1.1. Materials and Methods

5,5'-(((4,4,9,9-tetrakis(4-hexylphenyl)-4,9-dihydro-s-indaceno[1,2-b:5,6-b']dithiophene-2,7-diyl)bis(thiazole-2,5-diyl))bis(methaneylylidene))bis(1,3-diethyl-2-thioxodihydropyrimidine-4,6(1H,5H)-dione) (IDTz-BARS), and 5,5'-(((4,4,9,9-tetrakis(4-hexylphenyl)-4,9-dihydro-s-indaceno[1,2-b:5,6-b']dithiophene-2,7-diyl)bis(thiazole-2,5diyl))bis(methaneylylidene))bis(1,3-dimethylpyrimidine-

2,4,6(1H,3H,5H)-trione) (IDTz-BARO) were synthesized in this work. (4,4,9,9-tetrakis(4-hexylphenyl)-4,9-dihydro-s-indaceno[1,2-b:5,6-b']dithiophene-2,7-diyl)bis(tributylstannane) ($SnBu_3$-IDT-$SnBu_3$) and 2-bromothiazole-5-carbaldehyde were purchased from SunaTech Inc.. All other reagents were purchased from www.tansoole.com and were used as received without further purification. ^{1}H nuclear magnetic resonance (NMR) spectra were measured using a 300 MHz spectrometer (Varian Mercury Plus). Elemental analysis was characterized by （ELEMNTAR vario El cube）Thermal analyses (TGA) was performed using METTLER TOLEDO TGA2 (TA Instruments) and thermal analyses (DSC) were performed using NETZSCH DSC214Polyma (TA Instruments) under an inert N_2 atmosphere, with heating/cooling rates of 10 °C min^{-1}. The ultraviolet–visible spectra were recorded using a UV-Visible spectrophotometer (UV-1780). Cyclic voltammetry experiments were performed with an electrochemical analyzer (CH Instruments) in acetonitrile solutions containing 0.1 M tetrabutylammonium tetrafluoroborate (Bu_4NBF_4) as the supporting electrolyte, with Ag/$AgNO_3$ as the reference electrode, a platinum wire as the counter electrode, and a platinum working electrode. All the calculations are carried out with G09 ver. D software package. Geometry optimizations were carried out with DFT methods B3LYP for geometry. Basis set 6-31G(d) was adopted for the C, H, O, N, S and F atoms. Atomic force microscopy (AFM) measurements are performed by using a Dimension Icon Scanning Probe Microscope (Asylum Research, MFP-3D-Stand Alone) in tapping mode. The performance of the organic photovoltaic cell (OPV) was confirmed using a solar simulator (ss-x50, Enlitech). The current density-voltage (J–V) graphs of the devices were measured under AM 1.5G light conditions. Besides, the incident photon to converted current efficiency (IPCE) spectrum was measured using Newport IPCE system.

S1.2. Device fabrication

Bulk heterojunction OPV devices were fabricated with an inverted structure, consisting of indium tin oxide (ITO)/ZnO/active layer/MoO_3/Ag. Typically, the ZnO layer (~30 nm) acting as an electron transporting layer was coated onto ITO (pre-

cleaned with UV-ozone plasma), as previously reported. The active layer, which was prepared as follows: in chlorobenzene (total concentration = 23 mg·mL^{-1}), was fabricated by spin-coating at 3000 r/min for 30 s in a glove box filled with nitrogen. After drying the active layer, MoO_3 (~10 nm) and Ag (~100 nm) layers were formed by thermal evaporation under a base pressure of 2×10^{-4} Pa . The effective area of the device was 0.04 cm^2.

S1.3. Synthesis of Compound

S1.3.1. Synthesis of 5,5'-(((4,4,9,9-tetrakis(4-hexylphenyl)-4,9-dihydro-s-indaceno[1,2-b:5,6-b']dithiophene-2,7-diyl)bis(thiazole-2,5diyl))bis(methaneylylidene))bis(1,3-dimethylpyrimidine-2,4,6(1H,3H,5H)-trione) (IDTz-BARO)

A mixture of compounds 1 (90.37 mg, 0.08 mmol), 1,3-dimethylpyrimidine-2,4,6(1H,3H,5H)-trione (74.95 mg, 0.48 mmol), pyridine (0.5 ml) was stirred overnight in $CHCl_3$ at 65 °C. The reaction mixture was cooled to room temperature and concentrated under reduced pressure. The residue was purified by silica gel column chromatography and eluent (dichloromethane / petroleum ether = 1 / 1, v / v) to obtain a dark red solid product (50.56 mg, 45.0%). ^{1}HNMR results are as follows: ($CDCl_3$ 300MHz): ^{1}HNMR results are as follows: ($CDCl_3$ 300MHz): δ8.62 (s, 2H), 8.36 (s, 2H), 7.15 (d, 8H),7.06 (d, 8H), 3.14 (d, 12H), 2.56 (t, 8H), 1.57 (d, 8H), 1.28 (m, 64H), 0.86 (m, 24H). Element analysis for $C_{84}H_{88}N_6O_6S_4$, Calc.: C,71.76; H, 6.31; N, 5.98. Found: C, 72.08; H, 5.96; N, 5.79%.

S1.3.2. Synthesis of 5,5'-(((4,4,9,9-tetrakis(4-hexylphenyl)-4,9-dihydro-s-indaceno[1,2-b:5,6-b']dithiophene-2,7-diyl)bis(thiazole-2,5-diyl))bis(methaneylylidene))bis(1,3-diethyl-2-thioxodihydropyrimidine-4,6(1H,5H)-dione) (IDTz-BARS)

A mixture of compounds 1 (90.37 g, 0.08 mmol), 1,3-diethyl-2-thioxodihydropyrimidine-4,6(1H,5H)-dione (96.29 g, 0.48 mmol), pyridine (80.5 ml) was stirred overnight in $CHCl_3$ at 65 °C. The reaction mixture was cooled to room temperature and concentrated under reduced pressure. The residue was purified by

silica gel column chromatography and eluent (dichloromethane / petroleum ether = 1 / 1, v / v) to obtain a dark blue solid product (47.76 g, 40.0%). ^{1}HNMR results are as follows: ($CDCl_3$ 300MHz): δ8.61 (s, 2H), 8.38 (s, 2H), 7.72 (s, 2H),7.54 (s, 2H), 7.16 (d, 8H), 7.10 (d, 8H), 4.56 (dd, 8H), 2.57 (t, 8H), 1.57 (d, 16H), 1.26 (m, 64H), 0.86 (m, 24H). Element analysis for $C_{88}H_{96}N_6O_4S_6$, Calc.: C, 70.74; H, 6.48; N, 5.62. Found: C, 70.51; H, 6.56; N, 5.51%.

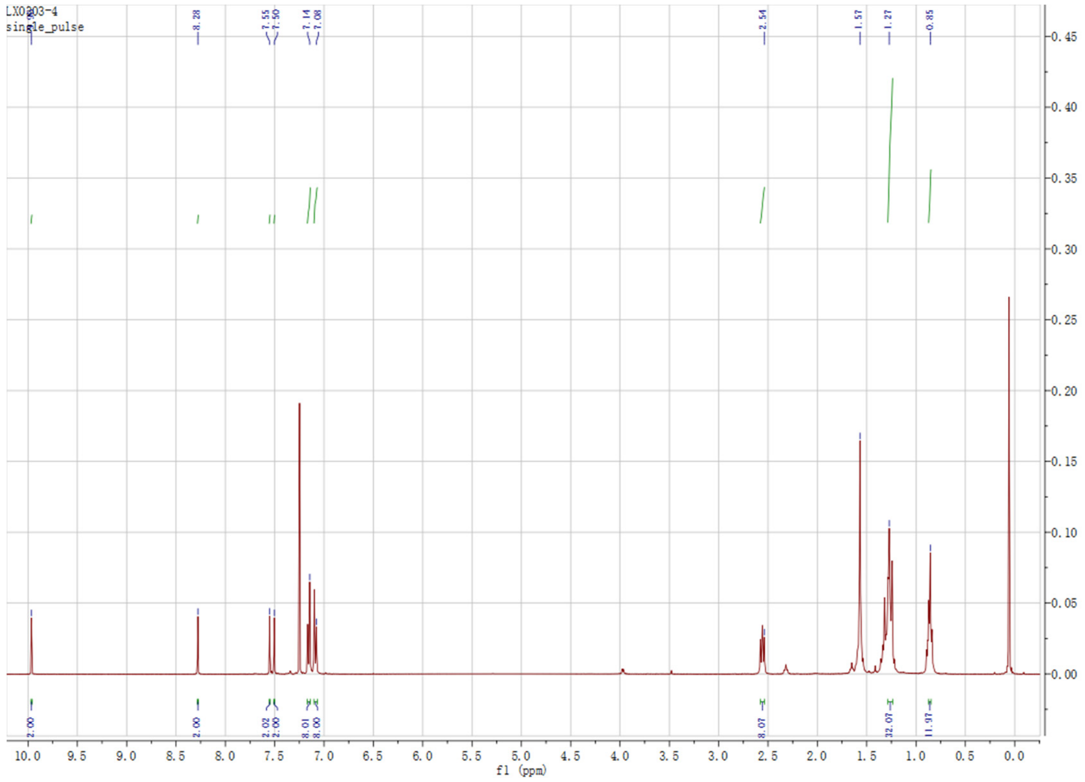

Figure S1. ^{1}H of compound 1. (r.t., in $CDCl_3$).

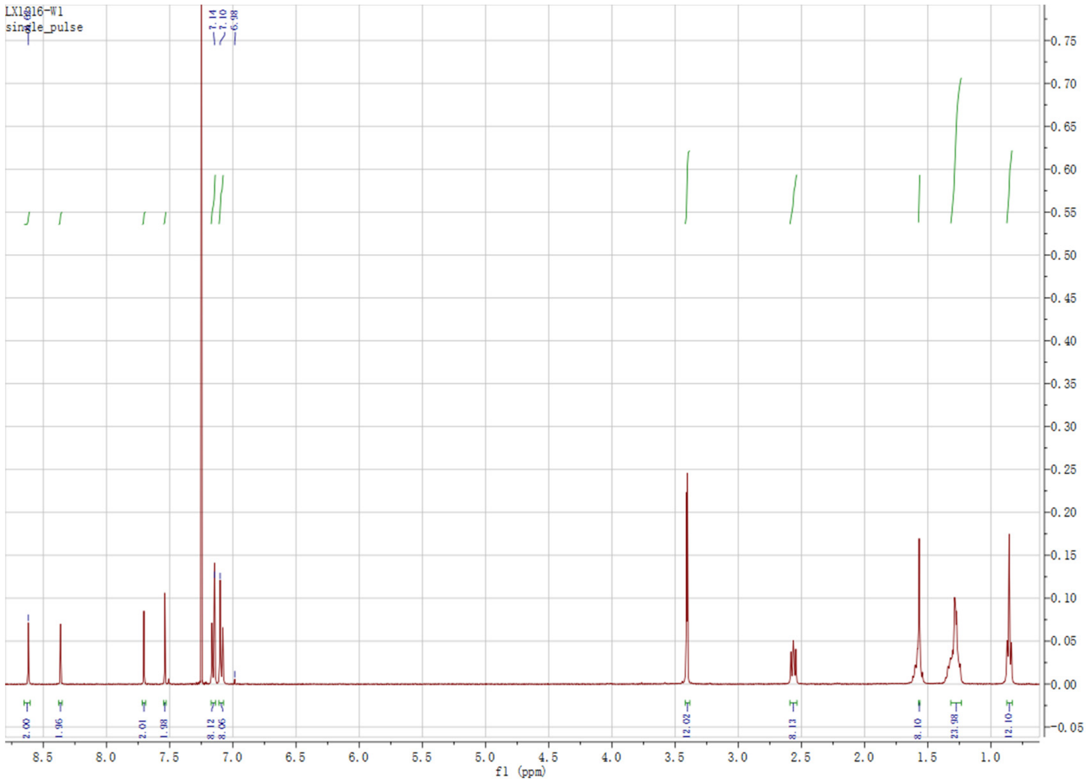

Figure S2. [1]H of compound IDTz-BARO. (r.t., in $CDCl_3$).

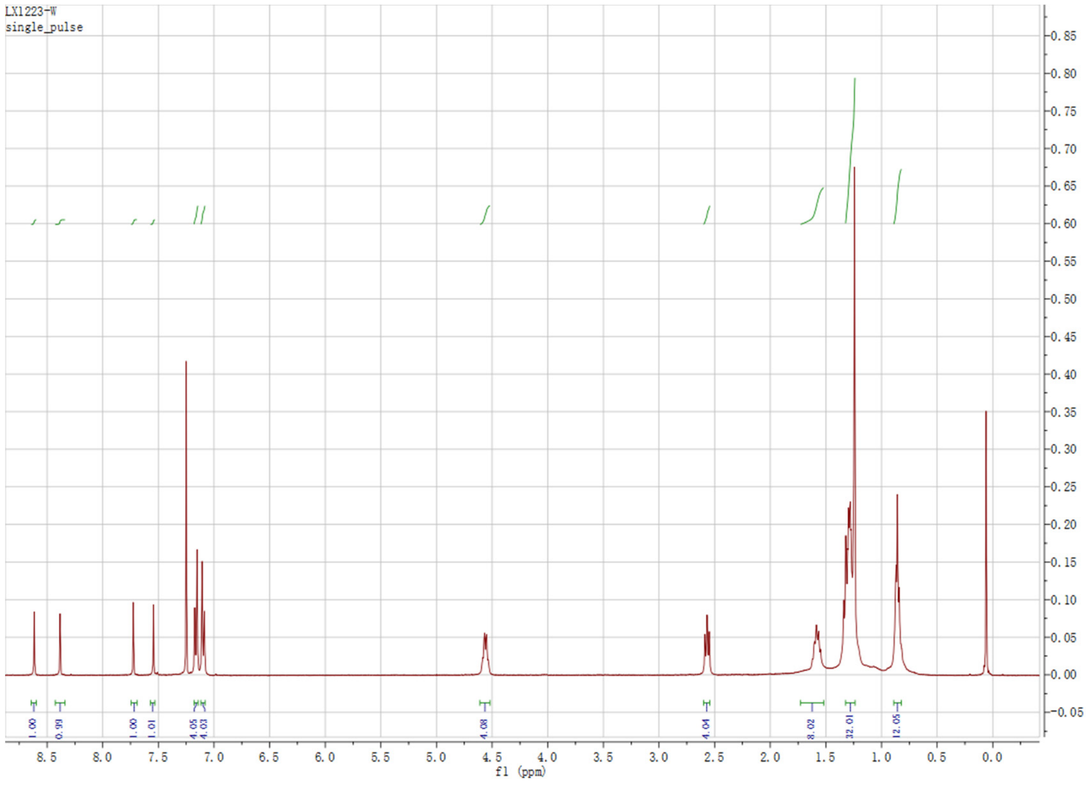

Figure S3. [1]H of compound IDTz-BARS. (r.t., in $CDCl_3$).
